# Utilization of a New Customizable Scoring Tool to Recruit and Select Pulmonary/Critical Care Fellows

**DOI:** 10.7759/cureus.15396

**Published:** 2021-06-02

**Authors:** Susanti R Ie, Jessica L Ratcliffe, Catalina Rubio, Kermit S Zhang, Katherine Shaver, David W Musick

**Affiliations:** 1 Pulmonary and Critical Care Medicine, Carilion Clinic, Roanoke, USA; 2 Medicine, Liberty University College of Osteopathic Medicine, Lynchburg, USA; 3 Internal Medicine, Virginia Tech Carilion School of Medicine, Roanoke, USA; 4 Biostatistics and Epidemiology, Carilion Clinic, Roanoke, USA; 5 Educational Evaluation and Policy Studies, Virginia Tech Carilion School of Medicine, Roanoke, USA

**Keywords:** fellowship selection, pulmonary critical care, education, assessment in health professions education, medical education & training, education and training of medical students and doctors (specialist and phd)), recruitment

## Abstract

Background: Finding the ideal candidate for a residency/fellowship program has always been difficult. Finding the “perfect” match has always been the ultimate goal. However, many factors affect obtaining that “perfect” match. In the past, we would have each attending physician review around 20 to 50 Electronic Residency Application Service (ERAS) applications and rank them into three categories: high, middle, or low. Depending on their ranking, the applicant would be invited for an interview. After the interview, the applicants’ files (ERAS and interview) would be reviewed and ranked by the faculty as a group. This was time-consuming and fraught with too much subjectivity and minimal objectivity. We, therefore, sought to find a way to assess and rank applicants in a more objective and less time-consuming manner. By creating a customizable scoring tool, we were able to screen applicants to our pulmonary/critical care fellowship program in an efficient and a more objective manner.

Objectives: A customizable scoring tool was developed weighting components in the ERAS and interview process, allowing residency/fellowship programs to create a final rank list consistent with the programs’ desired applicants.

Methods: Two hundred and sixty pulmonary/critical care fellowship applications were reviewed from 2013 to 2018. In 2018, we used our new scoring rubric to create a rank list and rescore previous applicants. The traditional and new lists were compared to the final rank list submitted to the National Residency Matching Program (NRMP) for 2018. We wanted to ascertain which scoring method correlated best with the final rank list submitted to the NRMP. We obtained feedback from eight faculty members who had reviewed applicants with both scoring tools.

Results: The novel customizable scoring tool positively correlated with the final rank list submitted to the NRMP (r= 0.86). The novel tool showed a better correlation to the final rank list than the traditional method. Faculties (6/6, 100%) responded positively to the new tool.

Conclusions: Our new customizable tool has allowed us to create a final rank list that is efficient and more focused on our faculty’s desired applicants. We hope to assess and compare the quality of applicants matched through this scoring system and the traditional method by using faculty evaluations, milestones, and test scores.

## Introduction

In 2019, it was noted that pulmonary/critical care medicine was the second most popular fellowship choice among the internal medicine specialties [1]. The pulmonary/critical care fellowship match is a highly competitive match, filling 99% of the available slots. In the US, there are 166 pulmonary/critical programs with 601 positions. Approximately 24.8% of applicants go unmatched [1]. With this being said, it is extremely important that each individual program select the ideal candidate that will fulfill the needs of both the program and the applicant. 

Applicants are initially reviewed through the Electronic Residency Application Service (ERAS) which was created by the Association of American Medical Colleges in 1995. The ERAS system contains a wealth of information about each applicant, including medical school, class rank, grades, residency programs, research experience, personal statement, and letters of recommendation. Our pulmonary/critical care program accepts four pulmonary/critical care fellows per year, and we receive and review approximately 300 applications each year. With the increasing dearth of critical care physicians, there has also been an increase in the number of pulmonary/critical care fellowship programs and positions available in the United States. Reviewing each individual applicant in sufficient detail is a formidable challenge for busy clinical faculty members.

We have sought a way to evaluate prospective applicants that would be less time-consuming, yet more aligned with what our fellowship program desires in an applicant. We have focused solely on objective measures when selecting candidates, as many additional factors are at play including the contents of personal statements which shed light on applicants’ reasons for pursuing a particular field and the life experiences they have had to date. However, as important as it may be, the personal statement cannot stand alone when selecting an applicant; other variables must also be evaluated. Many programs have attempted to develop a screening process to find the ideal applicant for their programs. Screening processes have included an assessment of the undergraduate and medical schools from which the applicant graduated, class rank, GPA, research experience, letters of recommendation, personal statements, and interviews. Unfortunately, there has been no consistently validated means of screening applicants [2-10]. The ERAS Application Scoring Tool-Interview Scoring Tool (EAST-IST) Study is the only published article that recently attempted to develop a novel scoring tool for a pulmonary and critical care fellowship program in generating an institution-specific rank list using both the ERAS application and the interview [11].

We aimed to create a similar customizable ranking tool when selecting applicants into our pulmonary/critical care fellowship program in the hopes of making the process more expeditious and finding fellowship candidates who would be an ideal fit for our program. By focusing on specific characteristics that we felt were more important than others, we hoped to capture the particular candidate who we felt would be successful in our field and in our program.

## Materials and methods

**Figure 1:**
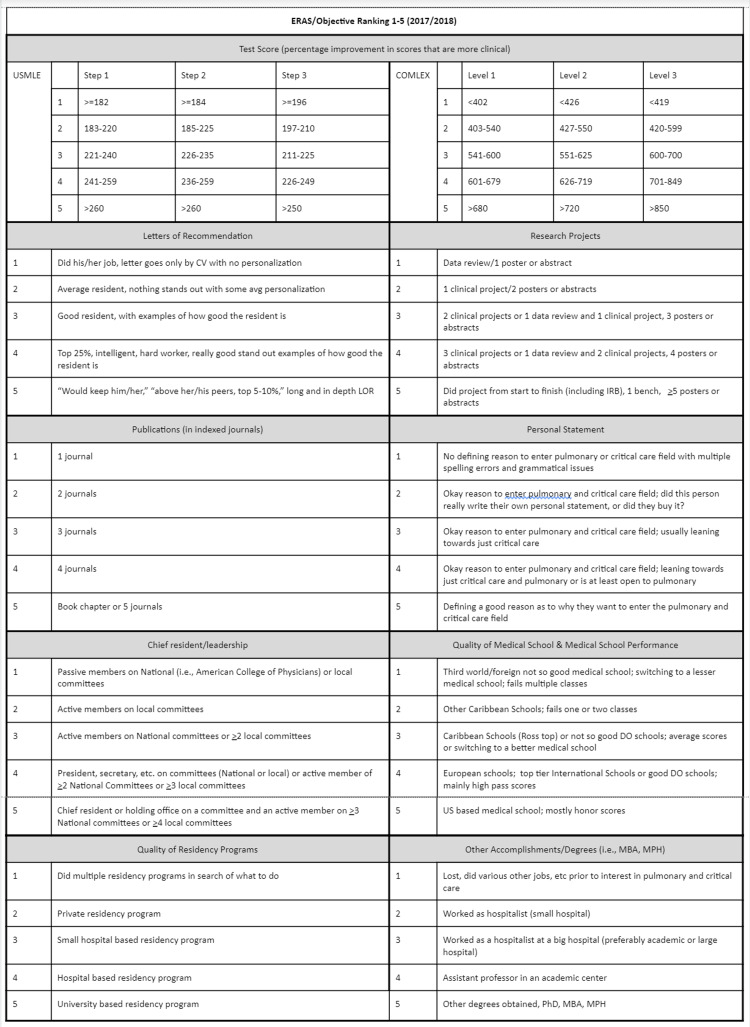
Pulmonary and Critical Care Fellowship Application Scoring Tool This is our system that we utilized to score the different elements found in an applicant’s ERAS file. Applicants are given a score from 1 to 5 for each section.

A new scoring rubric was developed whereby individual characteristics found on an applicant’s ERAS application could be weighed and prioritized depending on the preferences of the program. The scoring tool included scoring various attributes that are included in the ERAS application (e.g., United States Medical Licensing Examination [USMLE]/Comprehensive Osteopathic Medical Licensing Examination [COMLEX] score, letters of recommendation, research; Figure 1). There have been multiple studies trying to see if one single variable would predict how successful an applicant would be in a specific field, none have found that one single variable. Bosslet et al. studied multiple variables that were weighted and specific to an institution's desires that would help to select the ideal candidate for that institution [11]. Each attribute is weighed a certain percentage depending on what the faculty in the program feels is most important. As our program is mainly clinical, we look for applicants who are more clinically inclined with an interest in clinical research. As our program evolves, I suspect that the weights for the attributes will be adjusted. The rubric can be adjusted as your program expands, which is appealing. For example, the USMLE/COMLEX total score is weighted at 0.15. However, our program feels that the third component of the test is more clinically relevant than the first component and thus the weight for the first part is 0.03 and the final part is 0.07 (Table 1). In total, all the components in ERAS will have a cumulative raw score of 1 (maximum of 5), and this is 40% of the applicant’s composite score. In our rubric, the interview is weighted at 60% of the composite score (Table 2). For the interviews, we had one set of interviewers who ask the applicants questions regarding critical thinking and research, while another set of interviewers asked questions regarding work ethic and multitasking. Our program chose to score the interview process more than the components in the ERAS application due to the fact that the faculty felt they would be able to tease out subtle aspects of the applicants who were not easily identified in ERAS. If one was invited for an interview, then the applicant could either move up or down on the list depending on how they performed on the interview.

**Table 1:** Institutional Pulmonary and Critical Care Fellowship Scoring Rubric for Interview Consideration This is our scoring rubric that we used to match our fellows in 2018. Each component has a weighted score. Notice that the board exams in our figure demonstrate an increase in value with each subsequent examination due to our program's belief that the later exams test more relevant clinical skills and thought processes relevant to our discipline.

Name of applicant			
Medical school and program graduation year			
Residency program			
US citizen or VISA			
Name of reviewer			
Criteria	Score (min 1–max 5)	Weight (100%)	Weighted score (score × weight)
USMLE or COMLEX scores		0.15	
Step 1		0.03	
Step 2		0.05	
Step 3		0.07	
Letters of recommendations		0.1	
Research projects		0.075	
Publications		0.15	
Personal statement		0.025	
Chief resident/leadership		0.1	
Quality of medical school		0.15	
Quality of residency program		0.15	
Other accomplishments/degrees, i.e., MBA, MPH		0.1	
Cumulative raw score (max 60)	0	1	
Cumulative weighted score (max 5)			

**Table 2:** Sample New Rubric Rank List In Comparison to Submitted Rank List An example of the New Rubric Rank list (ERAS score + interview score) compared to the final rank list that is submitted to the NMRP.

ERAS Score	Interview Score	Total Score (40% ERAS Score + 60% Interview Score)	New Rubric Rank List	Final Rank List
4.08	5	4.63	1	1
3.6	5	4.44	2	2
3.78	4.75	4.36	3	3
3.31	4.75	4.17	6	4
3.87	4.63	4.32	4	5
2.83	4.4	3.77	26	6
3.53	4.5	4.11	7	7
3.64	4.38	4.08	8	8
3.82	4.25	4.08	9	9
3.61	4.38	4.07	10	10

For the past few years as our fellowship has continued to flourish, our pulmonary/critical care fellowship program has received approximately 300 applicants a year to review through ERAS. From 2013 to 2018, approximately 260 applicants were interviewed. Our new scoring rubric was first utilized in 2018 to both invite applicants for interviews and to create a rank list for 2018. All applicants from the previous recruitment years, going back to 2013, were rescored using this new rubric and a new rank list was created for each year from 2013 to 2017. Three rank lists were compared: the traditional list, the new list, and the final list submitted to the National Residency Matching Program, NRMP. By comparing these lists, we were able to ascertain which method correlated best with the final list submitted to the NRMP. Lastly, we surveyed six faculty members involved in the interview process from 2013 to 2018 obtaining their feedback on the new versus the traditional scoring system.

The study was exempt from Carilion Clinic IRB under the Department of Health and Human Services (DHHS) regulatory categories 2 and 4.

## Results

**Figure 2:**
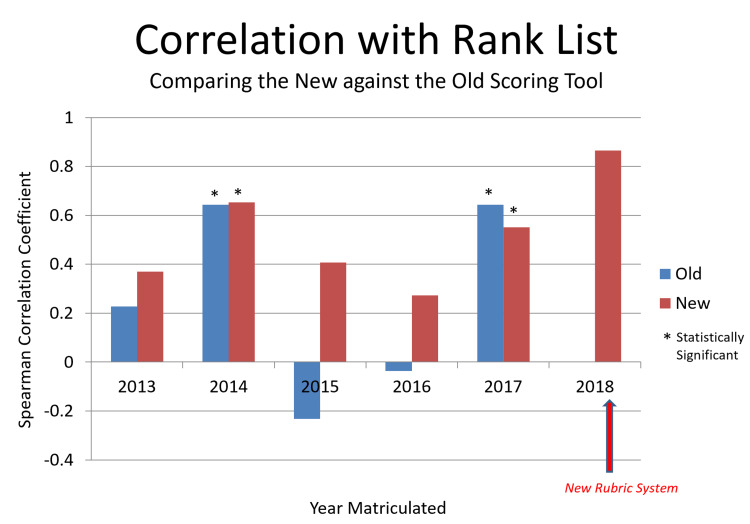
Comparison Between Applicant Scoring Rubrics in Correlation with Final NRMP Ranking Correlation of the old and new scoring tools used by our program with the final rank list that was submitted to the NRMP between 2013 and 2018.

The newly developed customizable scoring tool used in 2018 had a good correlation with the final rank list that was submitted to the NRMP (Spearman Correlation Coefficient, r= 0.86; Figure 2). Furthermore, from 2013 to 2017, the novel tool showed improved correlation to the final rank list when compared to the traditional method with the correlation being strongest in 2014 and 2017 (Spearman Correlation Coefficient, r: old vs new: 2013: 0.22756 vs 0.36985, p-value 0.3085; 2014: 0.64347 vs 0.65324, p-value 0.0002; 2015: 0.23320 vs 0.40711, p-value 0.2963; 2016 −0.03636 vs 0.27273, p-value 0.9155, and 2017: 0.64540 vs 0.55054, p-value 0.0012). The traditional rank list when compared to the new rank list varied tremendously in 2015 when compared to other years as that year had the most subjectivity when ranking applicants (neither the traditional or new ranking tool met clinical significance). Our rank list process allows an applicant to move up and down the list if a faculty member has strong feelings about an applicant’s potential capabilities and if all the other faculty members agree. The 2014 and 2017 results showed that both the traditional and new ranking tools had the most correlation with the final submitted rank list (both were highly correlated and clinically significant). These data support the use of the new ranking tool as being equally as efficacious and more efficient than the traditional ranking tool. Six out of six (100%) faculty members surveyed reported positive responses regarding the new scoring tool. One response stated that “the scoring system was improved, providing a more uniform scoring pattern amongst interviewers.”

## Discussion

The application process into most medical specialties has become increasingly competitive leading to an increasing burden when reviewing applications. This process is not just isolated within the medical community. The process for student selection in graduate programs affects these individuals’ success in courses, retention, and rates of graduation [5]. Similarly, the selection process for fellowship programs can heavily impact the fellows selected.

We developed a novel scoring tool based upon multiple candidate domains to screen and rank our applicants. The results of this study showed that this tool can be used to efficiently rank applicants based upon the factors that a given program feels are the most significant to their needs. Because the scoring tool generated a ranked applicant list that correlated positively with the final rank list submitted to the NRMP, the tool can be considered to be accurate in creating a list that is in line with what each individual program desires in its applicants. Additionally, we found that the correlation between the new list and the final rank list submitted to the NRMP was higher than the correlation between the old list generated by the traditional method and the final rank list submitted to the NRMP. Overall, this tool can allow for programs to create reliable rank lists by using criteria that are important to that particular program.

There are several limitations that exist in our study that could be addressed in future studies. Most importantly, comparing the new rubric to the traditional method does not measure the quality of resident/fellow that the program ultimately acquires. Future studies looking at milestones, tests, evaluations, and eventual career choices will allow programs to select applicants which represent what the program desires. Another limitation of our study would be the small number of participants who were evaluated due to the size of our fellowship program. This issue can be overcome by inviting other programs to utilize the customizable scoring tool and/or allowing time to accrue more participants as residents/fellows enter and leave the program. Additionally, surveying faculty members for evaluation of the tool was problematic as we could only survey six faculty members who have used both the old and new scoring tools due to the flux in faculty members over six years. One of the main values of the tool is that it saves time for the faculty and allows applicants to be reviewed in a more efficient and effective manner. It has also allowed the program to remove some subjective bias from the review process, allowing more consistency when reviewing applications.

Finding the ideal candidate for a program goes both ways. Not only does the program need to find the ideal candidate, but the candidate also has to find his or her ideal program. According to a 1995 study by Sklar and Tandberg, self-rank is strongly correlated with dean’s letter information and thus holds the potential to be used during early applicant screening, prior to receiving dean’s letters [12]. In the study, the applicants would self-rank themselves by providing an assessment of their relative standing in regard to their classmates. Additionally, there is a positive correlation between self-rank and final rank in the National Resident Matching Program match. Since the study by Sklar and Trandberg regarding an applicant’s self-rank in screening an applicant, there have been no further data supporting or negating this. Perhaps, because of the strong correlation that the self-rank has with the dean’s letter, it may have been felt that the dean’s letter was a sufficient variable in ranking an applicant. There is also evidence supporting the benefits of pre-interview dinners for both program candidates and fellowship programs themselves, as these dinners can help elucidate information about the programs to the candidates and aid in decisions between different programs, thus allowing candidates to select the program best suited to them [13].

As we continue to use our customizable scoring tool for our fellowship program, we will need to continue to assess and likely adjust the weights on each individual domain. As mentioned previously, finding novel ways to assess applicants could assist programs in their search for the ideal candidate. Medical schools have incorporated multiple mini interviews (MMIs) to see how applicants would deal with certain situations [14]. Trying to assess an applicant’s procedural skills through a simulation lab could also be worthwhile. One such study incorporated a nontechnical simulation (which included cognitive and interpersonal skills) with a personality test and showed that this multi-station selection process was feasible and had face validity. However, this process required the involvement of a high number of faculty [15]. Both the MMIs and assessing actual skills could be quite an undertaking as both would require a large investment of time.

With the COVID-19 pandemic and the importance of social distancing, new ways of interviewing applicants will need to be explored. The Accreditation Council for Graduate Medical Education (ACGME) is currently exploring virtual interviews as a safe way to meet applicants. As our scoring tool places 60% weight on the interview of an applicant, the weight of the interview may need to be adjusted once we are able to assess how the virtual interviews are able to capture the true essence of an applicant. As virtual interviews save costs not only for the programs but also for the applicants, these interviews or at least a hybrid of virtual/in-person interviews may become the standard for the future. Nevertheless, prior to matching the applicants, this could be problematic for both sides as the program would not physically meet the applicant until they start the residency/fellowship. Additionally, the applicant would not get to tour or meet the current residents/fellows in their future program, thus not allowing both sides the opportunity to better assess if they would be a good fit. Only time will allow us to see if virtual interviews are as efficacious as real-life interviews.

We plan to assess the quality of the applicants who we have matched through the traditional method and the new customizable scoring tool by comparing their evaluations from faculty members, test scores, and milestones. As our desire for certain qualities in our applicants evolves, we can adjust the percentages and even elements in the rubric thus customizing the rubric to what we desire in our fellows. We plan to evaluate the fellow’s milestones and review internal surveys to see if our program was an ideal match for the fellow and the fellow an ideal match for the program

Interestingly, there is an online company created by a pediatrics/anesthesiology resident and an anesthesiology program director who is also currently an associate designated institutional official (DOI) who formed a company named Thalamus. This company creates an online platform that helps both the program and the trainee with the application and interview process. They state on their website that they can assist the program with scoring and ranking applicants by creating scoring algorithms by measuring a candidates’ fit with a program.

## Conclusions

Ranking applicants to fellowship programs remain both challenging and time-consuming. Additionally, different programs vary in the indicators they choose to rely on and how those indicators are weighted. In this study, we developed a customizable scoring tool to rank applicants to our fellowship program by creating a composite score for each applicant. This tool weighted and utilized components in ERAS and the interview process in order to create a final rank list that remained consistent with the desired applicants for our residency/fellowship program.

Our results confirmed that this new customizable scoring tool has allowed us to create a final rank list that is helpful in narrowing the applicant list to our faculty’s desired applicants. In regard to the new scoring tool, a faculty member said, “There is a slight improvement, still hard to judge the applicants based on a single interview.” In other words, although designing scoring tools is vital and improves the application process, further research should focus on expanding ways of judging applicants apart from interviews or on improving the interview process. Although this study is a small/pilot study, we feel that the rubric has potential in selecting applicants applying into a residency/fellowship program. Only with time and likely with many modifications to the rubric, will we know how useful this tool can be. There are multiple parts of the application process that can be improved upon and this is just a start. We hope to share this customizable scoring rubric with other programs to create a more efficient and effective way to manage the residency/fellowship application process.
